# A refined method for theory-based evaluation of the societal impacts of research

**DOI:** 10.1016/j.mex.2020.100788

**Published:** 2020-01-23

**Authors:** Brian M. Belcher, Rachel Davel, Rachel Claus

**Affiliations:** a Sustainability Research Effectiveness Program, College of Interdisciplinary Studies, Royal Roads University, Victoria V9B 5Y2, Canada; b Center for International Forestry Research, P.O. Box 0113 BOCBD, Bogor 16000, Indonesia

**Keywords:** Theory-based Outcome Evaluation, Research evaluation, Impact assessment, Evaluation tools, Theory of change, Transdisciplinary research, Sustainability science

## Abstract

With high and increasing expectations for research to have social and environmental impact, there is a corresponding need for appropriate methods to demonstrate (for accountability) and analyze (for learning) whether and how research projects contribute to change processes. Evaluation is especially challenging for problem-oriented research that employs inter- and transdisciplinary approaches and intervenes in complex systems, where experimental and statistical approaches to causal inference are inappropriate. Instead, theory-based evaluation can be applied to identify and test causal processes. This paper presents a detailed explanation of the Outcome Evaluation approach applied in Belcher et al. (2019b). It draws on concepts and approaches used in theory-based program evaluation and the more limited experience of theory-based research evaluation, providing a brief overview of conceptual strengths and limitations of other methods. The paper offers step-by-step guidance on application of the Outcome Evaluation approach, detailing how to: document a theory of change; determine data needs and sources; collect data; manage and analyze data; and present findings. This approach provides a clear conceptual and analytical framework in addition to actor-specific and impact pathway analyses for more precision in the assessment of outcomes.

Specifically, the Outcome Evaluation approach:

• Conceptualizes research within a complex system and explicitly recognizes the role of other actors, context, and external processes;
• Utilizes a detailed actor-centred theory of change (ToC) as the analytical framework; and
• Explicitly tests a set of hypotheses about the relationship between the research process/outputs and outcomes.

## Graphical abstract

**Figure:**
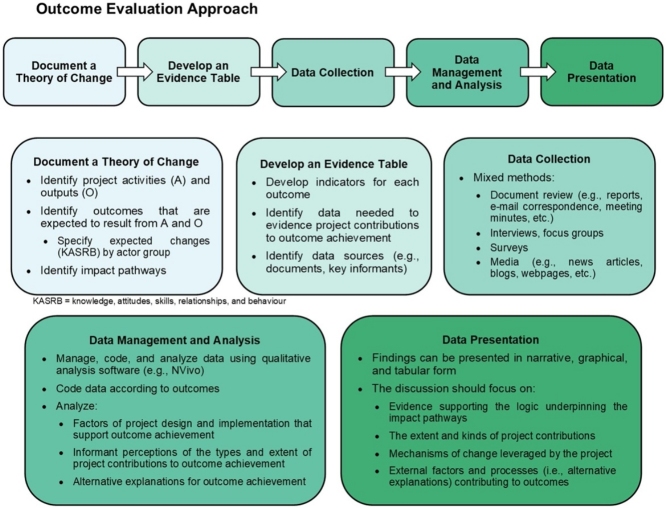

**Specification Table**

**Table:** 

Subject Area:	Environmental Science
More specific subject area:	Research Evaluation
Method name:	Theory-based Outcome Evaluation • Tool: Theory of Change (ToC)
Name and reference of original method:	Outcome Mapping (OM): • S. Earl, F. Carden, T. Smutylo, Outcome Mapping: Building Learning and Reflection into Development Programs. Ottawa: International Development Research Centre, 2001. RAPID Outcome Assessment: • ODI. RAPID Outcome Assessment, (2012). Retrieved from https://www.odi.org/sites/odi.org.uk/files/odi-assets/publications-opinion-files/7815.pdf Payback Framework: • M. Buxton, S. Hanney, How can payback from health services research be assessed? *J. Health Serv. Res. Pol., 1*(1) (1996) 35–43. Social Impact Assessment (SIAMPI): • J. Spaapen, L. van Drooge, Introducing ‘productive interactions’ in social impact assessment. *Res. Eval., 20*(3) (2011) 211–218. Contribution Analysis (CA): • J. Mayne, Addressing attribution through contribution analysis: Using performance measures sensibly. *Can. J. Program Eval., 16*(1) (2001) 1–24. • J. Mayne, Contribution analysis: Coming of age? *Evaluation, 18*(3) (2012) 270–280.
Resource availability:	Theory of Change: • Overview • B. Belcher, R. Claus, R. Davel, S. Jones, L. Ramirez, Research Theory of Change: A Practical Tool for Planning and Evaluating Change-oriented Research, (2019a). Retrieved from https://researcheffectiveness.ca/wp-content/uploads/sites/7/2019/08/Theory-of-Change-Toolkit.pdf • Network of Transdisciplinary Research (td-net). (n.d.). Theory of Change. Retrieved from https://naturalsciences.ch/topics/co-producing_knowledge/methods/td-net_toolbox/theory_of_change • Sample Facilitating Questions • Sustainability Research Effectiveness. (2019). Theory of Change Workshop – Building Your Theory of Change: Facilitating Questions. Retrieved from https://researcheffectiveness.ca/wp-content/uploads/sites/7/2019/02/Theory-of-Change-Facilitating-Questions.pdf • Model Templates • Sustainability Research Effectiveness. (2018b). Spheres Theory of Change Template. Retrieved from https://researcheffectiveness.ca/wp-content/uploads/sites/7/2018/10/spheres-Theory-of-Change-template.docx • Sustainability Research Effectiveness. (2018c). Traditional Theory of Change Template. Retrieved from https://researcheffectiveness.ca/wp-content/uploads/sites/7/2018/10/traditional-Theory-of-Change-template.docx • Evidence Table Template • Sustainability Research Effectiveness. (2018a). Evidence Table Template. Retrieved from https://researcheffectiveness.ca/wp-content/uploads/sites/7/2018/09/Evidence-table-template.doc Qualitative Analysis • Example Guiding Evaluation Questions (Appendix 1) • Example Key Informant Interview Guide (Appendix 2) • NVivo (data analysis software) • https://www.qsrinternational.com/nvivo/what-is-nvivo • Example Codebook (Appendix 3)

## Background

There are high and increasing expectations from research funders, society, and researchers themselves for research to have positive social and environmental impact, and there is a commensurate need for appropriate research evaluation methods for both accountability and learning. Assessing the societal impacts of research is more difficult than assessing advances in knowledge, and evaluating research that crosses disciplinary boundaries (interdisciplinary research) and actively engages with societal actors as part of the research process (transdisciplinary research (TDR)) to influence policy and practice is especially challenging [[1], [2], [3], [4], [5]]. Research of this kind interacts with multiple actors along multiple impact pathways by design. Each case is unique, and it is very difficult (if not impossible) to identify a counterfactual comparator. Therefore, experimental and statistical approaches to causal inference are not appropriate [6]. Instead, theory-based evaluation can be applied to identify and test causal processes.

There is a long history of theory-based evaluation [[7], [8], [9], [10], [11], [12]]. Though theory-based approaches have been applied mainly to program evaluation, there are a number of notable advances in theory-based research evaluation, including the Payback Framework [13] and Contribution Analysis (CA) [5,14,15], each with their own strengths and limitations. We have drawn on these and other program and research evaluation approaches (e.g., [6,[16], [17], [18], [19], [20], [21]]) to develop a method to assess research contributions in complex systems. The approach assesses whether and how a research project contributed to the achievement of outcomes, using the project’s theory of change (ToC) as the analytical framework and empirically testing it [[22], [23], [24], [25]]. This paper presents a detailed explanation of the Outcome Evaluation approach applied in Belcher et al. [25]. In this paper, we will describe how the method builds upon and differs from other theory-based evaluation approaches. We will then present details of the Outcome Evaluation approach, with step-by-step guidance on how to apply the method and effectively present the results. We will conclude with comments on method limitations and reflections of our experiences of applying the method.

## Method details

We employed a theory-based evaluation approach for each of the five outcome evaluations presented in Belcher et al. [25]. The approach uses a ToC as the key conceptual and analytical framework [8,[22], [23], [24],^26^,^27^]. A ToC aims to provide a comprehensive description and illustration of how and why a desired change is expected to occur in a specific problem context [28]. It models the causal relationships between a project’s activities and results and how these are expected to manifest in outcomes, giving particular attention to the impact pathways, actors, and steps involved in the change process. The approach explicitly recognizes that socio-ecological systems are complex and causal processes are often non-linear [29]. A ToC sets out testable hypotheses of a change process by working back from long-term goals to identify all the conditions that theoretically must be in place for the intended high-level results to occur. It is then possible to identify and collect the necessary evidence to assess actual achievements against expected outcomes at each stage.

### Relevant antecedent evaluation concepts and methods

There are many program evaluation methods and some specific research evaluation methods that have informed our approach. The most relevant methods are briefly described here, with a focus on key concepts and limitations of the methods for application in use-oriented research evaluation/impact assessment. Table 1 presents a comparison of other theory-based evaluation methods and the Outcome Evaluation approach.

**Table 1:** Comparison of theory-based evaluation methods.

Method	Research-specific	Uses Theory of Change	Level of Assessment	Type of Change Assessed	Actor-specific Outcome Framing	Tests Alternative Explanations
Outcome Mapping	No	Yes	Outcomes	Behaviour	Yes	No
RAPID Outcome Assessment	No	No	Outcomes	Behaviour	Yes	Yes
Payback Framework	Yes	Yes	Outcomes, impacts	Knowledge	No	No
SIAMPI	Yes	No	Outcomes	Knowledge, behaviour	Yes	No
Contribution Analysis	No	Yes	Outcomes, impacts	Behaviour	No^a^	Yes
Outcome Evaluation Approach	Yes	Yes	Outcomes, impacts	Knowledge, attitudes, skills, relationships, behaviour	Yes	Yes

a Originally, contribution analysis did not frame ToC by actor; however, Koleros and Mayne [39] propose the use of actor-based ToCs to help “unpack complexity” (p.293).

#### Outcome Mapping

##### Use

Outcome Mapping (OM) was developed as a method for monitoring and evaluating progress in development projects [16].

##### Key concepts

OM offers five key concepts that have informed our approach. First is the explicit recognition that the relative influence of any project or program declines the further one moves from the project boundary. In other words, while project managers may have a high level of control over project activities and outputs, they can only expect to have influence (not control) where project outputs interact with other actors and processes. This idea is conceptualized in three concentric spheres: the sphere of control; the sphere of influence, where the project still exerts some direct or indirect influence; and the sphere of interest, which includes the higher-level project aims, but falls outside the project’s sphere of influence. Second is the focus on results (outcomes) that are proximate to the intervention and occur within the sphere of influence. Outcomes result downstream from the initiative’s outputs, but upstream from longer-term political, demographic, economic, or environmental changes (impacts). This shifts emphasis from ‘impacts’ to ‘outcomes’ as the appropriate level for projects to target change and for evaluations to assess influence. Third, OM emphasizes that most change will result through the actions of others and that the project can be most effective by trying to influence actors at the project boundary (so-called ‘boundary partners’) to contribute to and support higher level objectives in the sphere of interest. OM includes a range of analytical tools and tactics for engaging, supporting, and otherwise influencing the actions of boundary partners. Fourth, outcomes are defined as changes in the behaviour of boundary partners. Finally, OM uses progressive indicators of observable behaviour change (‘progress markers’ in OM terminology) per boundary partner to demonstrate progressively deepening transformation. These are framed in terms of what one might realistically “expect to see” ([16], p.54) if a project is successful; what one would ideally “like to see” (p.54) if progress is excellent; and what one would “love to see” (p.54), which might be an unrealistic but aspirational target.

##### Limitations for TDR evaluation

Although used mainly in evaluation of social development projects and programs, OM concepts and methods are applicable in research evaluation, especially for change-oriented research operating in a systems context. However, OM explicitly avoids focus on higher-level impacts, and the tools and methods available do not accommodate analysis of research contributions to that level. As with most tools and methods used primarily in a development context, it does not adequately deal with research projects where a main focus is knowledge production. The approach also does not account for counterfactuals, so a proxy is needed in lieu.

#### RAPID Outcome Assessment

##### Use

The RAPID Outcome Assessment approach, which is based on OM, was developed to map and assess project contributions toward targeted changes in policy or a policy environment [20]. It has been used to assess the policy influence of research (e.g., [30]). Like OM, it focuses on key actors directly influenced by the project and the progressive changes in those actors.

##### Key concepts

As in OM, the focus on behaviour change is useful in TDR evaluation, as TDR explicitly aims to influence a range of actors through direct engagement. RAPID Outcome Assessments also consider the contributions of external influences in a change process, which helps in determining the relative project contributions to outcome achievement. The participatory nature of the data collection and analysis, involving project participants and various stakeholders adds depth and – along with the explicit use of a timeline – attempts to assess the relative contribution of the project to actual changes.

##### Limitations for TDR evaluation

This method does not make explicit use of a ToC. The outcome stories are derived inductively, without the ability to test hypotheses. OM does not account for counterfactuals, so a proxy is needed in lieu.

#### Payback Framework

##### Use

The Payback Framework was originally developed to assess medical and health service research impacts [31], and was one of the first research evaluation tools to integrate assessment criteria relating to research outputs and societal impact. The framework has two core elements: 1) a logic model of the complete research process, conceptualized as seven distinct stages (research inception (stage 0) to final outcomes (stage 6); and 2) multi-dimensional categories of research ‘paybacks’ or benefits (i.e., knowledge, research benefits, policy and product development, health sector benefits, broader economic benefits) [13,32,33]. The Payback Framework considers both direct interfaces between the research process and intended users and indirect influences through the reservoir of knowledge [13,22]. The framework’s structure enables systematic data collection and cross-case analysis [13].

##### Key concepts

The logic model of the Payback Framework is an applicable tool for TDR evaluation to trace research contributions to outcomes. The underlying theory of the framework is conceptually useful, as it posits that research exerts influence by producing and sharing knowledge. The framework also proposes that a research project can have ‘impact’ at any stage in a policy cycle, from “the initial step of issue identification or at the final step of implementing a solution” ([34], p.205), by influencing stakeholder knowledge [22].

##### Limitations for TDR evaluation

The Payback Framework does not adequately deal with contributions other than knowledge (i.e., attitudes, skills, relationships). This limits the comprehensiveness of the evaluation’s conclusions, as knowledge may be less influential than social process contributions that enable knowledge production, uptake, and transformation [22,25,35]. While the model contains feedback loops, the underlying theory is based on a linear model of research uptake-use-scaling-impact. The approach also does not account for counterfactuals, so a proxy is needed in lieu.

#### SIAMPI

##### Use

The “Social Impact Assessment Methods for research and funding instruments through the study of Productive Interactions between science and society” (SIAMPI) aims to uncover and assess the interactions between researchers and stakeholders to trace research contributions to social impact [18,19]. Productive Interactions are defined as exchanges between researchers and stakeholders (i.e., direct/personal engagement, indirect interactions though text or artefacts, financial interactions) in which knowledge is produced and valued. The approach was developed to address the difficulties in measuring complex and non-linear social impacts of research, as there is a lack of robust, reliable, and accepted indicators and measuring instruments [19]. The approach assumes that contact between researchers and stakeholders is a prerequisite for social impact to occur [18,19]. When contact leads to one or more stakeholders engaging with, using, or applying the research, the interaction then becomes ‘productive’. There is not always a clear distinction between ‘productive interactions’ and social impact because the transition from interaction to impact is often gradual [19]. Social impacts are achieved when productive interactions lead to stakeholder behaviour change (i.e., initiating new actions, changing how existing actions are done), though not all productive interactions necessarily lead to impacts [18,19].

##### Key concepts

The SIAMPI approach, more than any other, highlights the importance of interactions between researchers and other actors in a system of change. Understanding this is fundamental to understanding key mechanisms by which TDR works. Understanding how interactions or engagement approaches with particular actors influence and contribute to the creation and use of knowledge, as well as other aspects of behaviour change, is critical to understanding whether and how research contributes to change. It also helps legitimize efforts by researchers to engage stakeholders, and directs evaluators to pay attention to those interactions.

##### Limitations for TDR evaluation

Notwithstanding the emphasis on engagement and interactions, the approach still tends to focus primarily on the transmission and use of researcher-generated knowledge and less on knowledge co-production, capacity development, relationship-building, and other results of engaged TDR. It also lacks the systematic analytical framework of theory-based approaches, and it does not attempt to assess the value or quality of the impact [18]. Molas-Gallart and Tang [18] recognize that not all impacts may result in a social benefit; either the impact is not socially relevant, or it is perceived to have negative effects. Spaapen and van Drooge [19] note that conflicting narratives may emerge when applying the approach; opinions or perceptions of how changes occur or whether an impact has positive or negative implications can vary by stakeholder. This can also be seen as a strength, but it highlights the need for careful interpretation by the evaluators to understand differing perspectives. SIAMPI also does not account for counterfactuals, so a proxy is needed in lieu.

#### Contribution Analysis

##### Use

Contribution Analysis (CA) was developed to assess social intervention performance by exploring the causes and effects of a project or program. The approach was not developed explicitly for research contexts but has been applied for research evaluation [36,37]. Assessing an intervention’s contribution to outcome achievement is challenging [14,17,38]. When an intervention is situated in complex contexts, with multiple actors and processes that affect outcomes in some way, the extent of the intervention’s actual attribution is ambiguous [14,17]. CA is used to demonstrate “‘plausible association’” (Hendricks 1996, as cited in [17], p.8), which ascertains an informant’s knowledge of the intervention, perceptions of outcome achievement, and whether the intervention contributed to the achievement of outcomes.

##### Key concepts

CA uses ToC deductively, with systematic testing of an intervention’s contributions toward outcomes. CA appreciates that change happens within a system, and can therefore accommodate complex transdisciplinary contexts. As part of this, CA recognizes that an intervention is likely to operate in conjunction with other factors and processes; the method supports the exploration of external influencing factors and alternative explanations to help test and analyze the contributions of the intervention under assessment. CA specifically aims to identify and test competing hypotheses for key changes, which helps address the lack of a counterfactual comparator.

##### Limitations for TDR evaluation

ToCs used in CA are developed retrospectively by the evaluator, mainly using project documentation. Without the active involvement of project personnel and other stakeholders, key intentions or connections may be missed. In addition, ToCs in published examples of CA tend to aggregate expected results and actors which can miss the specific changes by actor or actor group. A recent paper by Koleros and Mayne [39] addresses this with added emphasis on using actor-based ToCs in CA. CA does not account for counterfactuals, so a proxy is needed in lieu.

### Step-by-step guidance on the Outcome Evaluation Approach

**Fig. 1:**
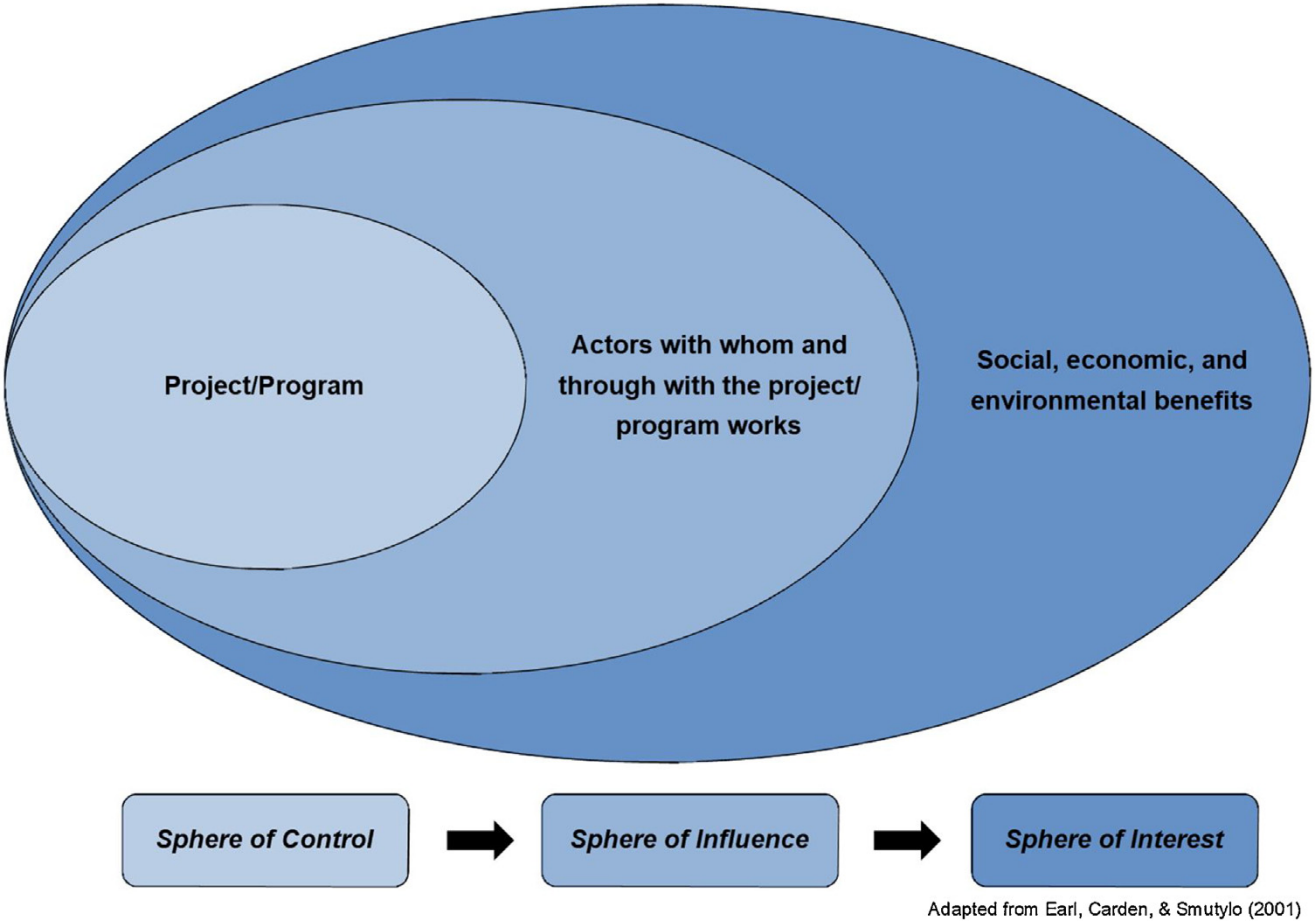
Influence declines as projects move from what they do (sphere of control) and who they work with and through (sphere of influence) to the improved conditions they hope to see (sphere of interest).

The Outcome Evaluation approach is explicitly intended to be applied to research projects and especially transdisciplinary research, sustainability research, research-for-development, and other change-oriented research approaches. It takes a systems perspective, acknowledging and appreciating that any project operates in conjunction with other actors and processes. It uses the OM concepts of declining relative influence conceptualized as spheres of control, influence, and interest (Fig. 1); outcomes defined as behaviour change; and specific indicators/measures defined per outcome. The underlying mechanisms of behaviour change are conceptualized (and emphasized) as changes in knowledge, attitudes, skills, and/or relationships (KASR), all of which can be influenced by the research project’s activities and outputs. An explicit and detailed project ToC is incorporated within the OM spheres and documented in a participatory way with project researchers, partners, and stakeholders.

As the intervention under evaluation is research, it is reasonable to expect that knowledge will be an important factor. Nevertheless, especially in a transdisciplinary context, there are many ways knowledge can be acquired and many other factors that can influence KASR and resulting action. We need nuanced and precise understanding of specific outcome achievements before generalizations can be made. See Appendix 1 for a generalized list of guiding evaluation questions that can help frame the research outcome evaluation and project assessment (n.b. these questions can be adapted to fit the scope and objectives of the evaluation).

A hallmark of the Outcome Evaluation approach is its specificity. For example, ‘knowledge products’ are specified in terms of the actual knowledge produced by the project, as opposed to simply listing publications or other media that communicate research-based knowledge. In other words, the approach can highlight what knowledge is now known that would not be known in the absence of the project. The approach also uses actor-specific outcomes. The method seeks to assess whether a research project has contributed to changes in KASR and behaviour of specific actors in the system. Outcomes should be framed in terms of who is doing what differently as a result of the project.

Ideally, a ToC would be articulated at the start of the project, but the method is designed to enable documentation and use of the ToC retrospectively. A key element of the approach is the explicit definition of end-of-project outcomes, defined as outcomes that would be ambitious but reasonable to expect within the timeframe and resources of the project being evaluated. Higher-level (i.e., beyond end-of-project) outcomes are also modelled in the ToC to illustrate and explain the causal logic to the impact level. The ToC is used explicitly as the main analytical framework to define data needed and identify potential data sources to test each change hypothesis deductively. The method employs an evidence table to organize and present data transparently and systematically by outcome. We build on the RAPID approach of eliciting participant and stakeholder perspectives (i.e., expert judgement) to identify and assess the relative contribution of various factors within a change process. The stakeholder interview is designed as a funnel, starting with broad questions about the change process and ending with specific questions about the research project. We also follow the CA approach of explicitly articulating and testing alternative hypotheses that may explain key changes.

A step-by-step description follows, providing detail on how to document a ToC, determine data needs and potential sources, collect data, manage and analyze data, and present findings.

#### Documenting a theory of change

A detailed project ToC serves as the analytical framework for an Outcome Evaluation. A ToC models the change process, providing a description and explanation of how and why the project was expected to contribute to a process of change as a set of testable hypotheses [29,40]. The ToC specifies the main actors involved in the change process and identifies their actions as a sequence of steps.

Often researchers have an implicit ToC on how they expect or intend for their project to contribute to real-world changes. In some cases, we have observed that members of the research team may have different implicit ToCs. By documenting a ToC, the Outcome Evaluation approach makes relationships between what a project does (activities and outputs) and what it aims to achieve (outcomes and impacts) explicit. This process can eliminate ambiguity or inconsistencies in understanding between members of the research team and collaborators, making project activities more cohesive and aligned.

Documenting a ToC is a participatory process, and the resulting model is owned by the research team. We recommend evaluators work closely with members of the project team and, if possible, other partners, collaborators, and stakeholders of the project to document the ToC. It is critical to have the project leaders participate in the workshop, but value is added when other stakeholders are involved for a breadth of perspective.

A ToC can be developed *ex ante* or *ex post* (for utility of ToC *ex ante*, mid-project, and *ex post*, see [29,40]). To date, all of the outcome evaluations we have done have used ToCs documented retrospectively, following the completion of the project; though we have also worked with numerous teams to develop ToCs at inception for research projects that are currently in progress. We recommend organizing a workshop with one or more facilitators to document the ToC with participants (i.e., the project team and other stakeholders). Ideally this would be conducted in-person, but it is possible to host a workshop online using video conferencing and screen-sharing software (e.g., BlueJeans, Zoom, Skype, Google+ Hangouts, etc.). Based on our experience, we recommend planning for a two-day workshop.

ToC documentation can be done within a set of nested spheres (Fig. 1), reflecting the declining relative influence of the project [16]:

*Sphere of Control*: A research project has a relatively high level of control over research definition, design, implementation (data collection and analysis), and the generation of outputs. Research contributions can come from any and all elements of the research process.

*Sphere of Influence*: Beyond the project boundary, there are many other actors and processes at work. The project cannot control what happens, but it can exert influence in many different ways. The degree of influence is likely to be highest closer to the project boundary, among direct partners, stakeholders, and users of research products and services.

*Sphere of Interest*: If research is successful at stimulating or contributing to change within the sphere of influence, it is reasonable to expect further changes. If key actors do something differently as a result or partially as a result of the research, that may in turn contribute to further changes. These changes will help transform systems and ultimately lead to social, economic, and environmental benefits.

There is no perfect way to develop a ToC, but experience suggests that it is helpful to begin by defining the overall purpose and then iteratively developing a model of the main activities, outputs, actors, outcomes, and impacts. Often some of these elements are articulated in the project proposal. We use specific definitions for each component of the ToC:

*Purpose*: The overarching goal the research aims to contribute to (but is not accountable for).

*Activities*: Actions conducted by the project or program (e.g., background scoping and preparation work, defining research questions, project design, literature review, fieldwork, planned communication and/or engagement with relevant stakeholders or boundary partners, etc.).

*Outputs*: The products, goods, and services of the research and the research process (i.e., knowledge, fora, and processes generated by the activities).

*Outcomes*: Changes in knowledge, attitudes, skills, and relationships manifested as changes in behaviour.

*Impacts*: Changes in flow (e.g., higher annual income, increased water discharge from a river) or state (e.g., socio-economic status, water quality in a reservoir), resulting wholly or in part from a chain of events to which the research has contributed.

*Results*: A collective term for outputs, outcomes, and impacts.

It is important to consider relevant actors and their role within the system where the project is operating, thinking how they could be involved in or influenced by the project activities and outputs. The essential question in developing the ToC is “who will do what differently as a result of the project?”. We recommend defining outcomes by specific actors or actor groups. Setting boundaries to define actors is contingent on the project context and the level of outcome. This helps delve into complex processes and facilitates the identification of data needs and potential data sources (i.e., informants) to test whether each outcome has been realized. We also recommend clearly differentiating between intermediate, end-of-project, and high-level outcomes. By considering when it is reasonable to expect a particular outcome to be realized or observed, hypothesizing the causal logic becomes easier and the testing of outcomes becomes more manageable and realistic.

*Intermediate outcome*: is observable during a project.

*End-of-project outcome*: is reasonable to expect within the timeframe and resources of the project and is observable at the conclusion of a project (and therefore testable during post-project evaluation).

*High-level outcome*: would be observed after the conclusion of a project; supports the causal logic if realized and provides evidence of a causal relationship from end-of-project outcomes to impacts (yet takes more time to manifest and is affected by more variables beyond the influence of the project).

We have compiled a set of facilitating questions [41] to help evaluators and research teams think through important components of the project ToC.

When all project activities, outputs, outcomes, and impacts have been identified, these components can be clustered around impact pathways that emerge during the process. Impact pathways are defined by the primary actors or actions to be influenced. For example, a policy pathway represents the constellation of actors and actions that would, in theory, contribute to a change in policy. The specificity of the approach by actor and impact pathway allows for more precision in the assessment of outcomes during the analysis stage.

The next step is to document assumptions that help explain why a particular change is expected to occur in a particular circumstance. In practice, we distinguish between two types of assumptions: 1) theoretical assumptions, which are the internal factors and/or mechanisms explaining why a change is expected (factors within the control of a project); and 2) contextual assumptions, which are the external factors and/or mechanisms explaining why a change is expected in a particular case (factors outside the control of a project). By making the assumptions explicit, they can be tested to inform learning about how a particular change manifests under the conditions of the project and the context in which the project is situated. Assumptions are documented in the evidence table (see Table 2).

**Table 2:** Example of an evidence table used to plan and track data collection.

Outcome (including assumptions)	Indicators	Data needed:	Data available: (what data exist, what data are already available)	Data sources: (data collection tool, list of informants)
	Expect to see:			
	Like to see:			
	Love to see:			
	Expect to see:			
	Like to see:			
	Love to see:			
	Expect to see:			
	Like to see:			
	Love to see:			

A ToC model is typically presented in the form of a flow diagram and accompanying narrative. The actual documentation of a ToC during the workshop can be done digitally or using pens and paper. Two digital ToC templates are provided: Fig. 2 sets the ToC within a set of nested spheres [42] while Fig. 3 uses a more traditional flow diagram [43]. We recommend using coloured boxes to differentiate between each stage of the ToC. The use of pens and paper on an expansive wall space can enable more participatory engagement of the workshop participants. Components of the ToC are mapped in real-time, and validated with participants to ensure accuracy. In the ToC workshops that we have hosted, one facilitator leads the brainstorming and documentation of the project ToC on the wall while a second facilitator documents the ToC digitally. We have found this approach to be efficient with limited time and participant availability.

**Fig. 2:**
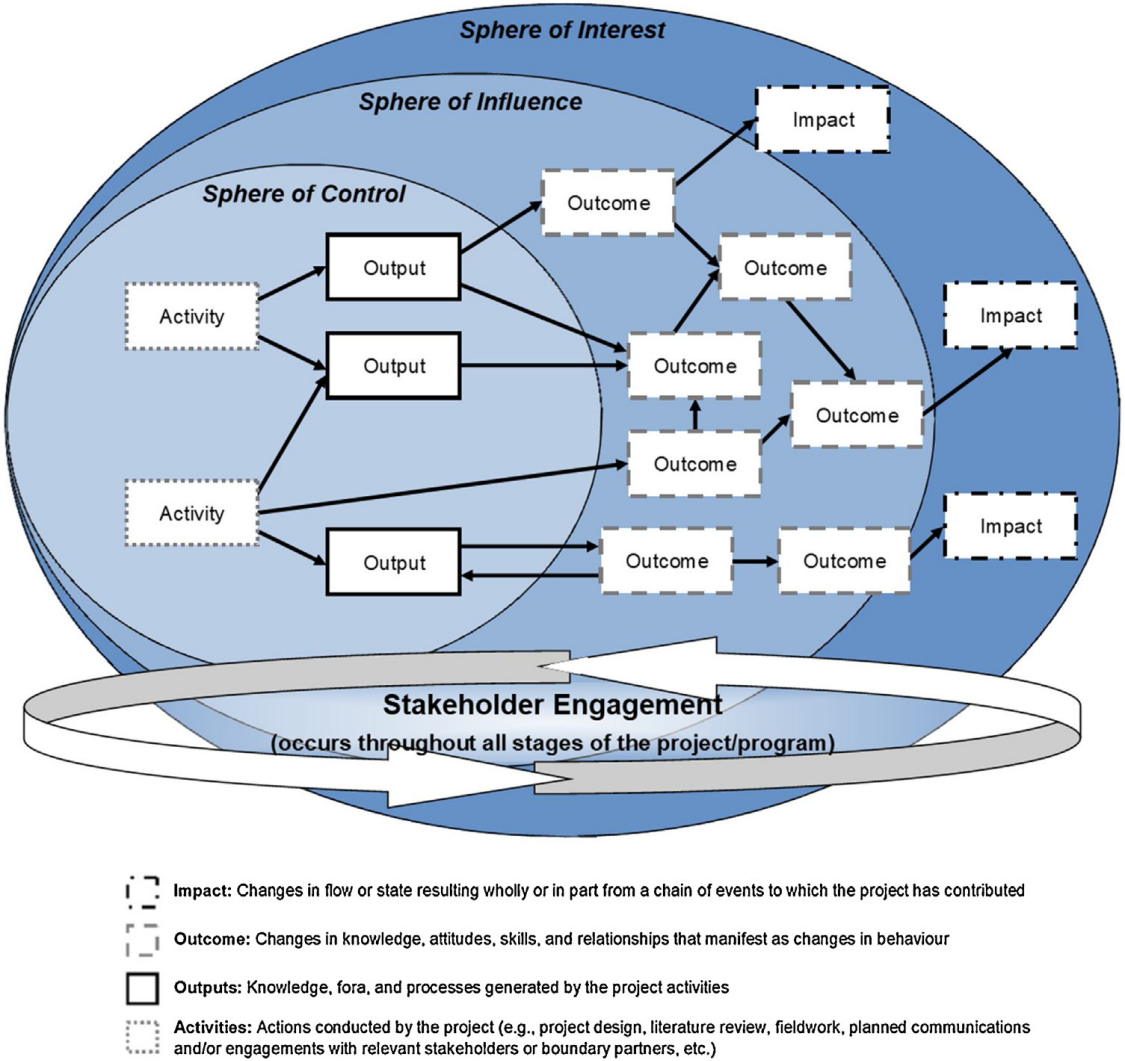
Sample theory of change spheres diagram demonstrating logical flow of activities, outputs, outcomes, and impacts.

**Fig. 3:**
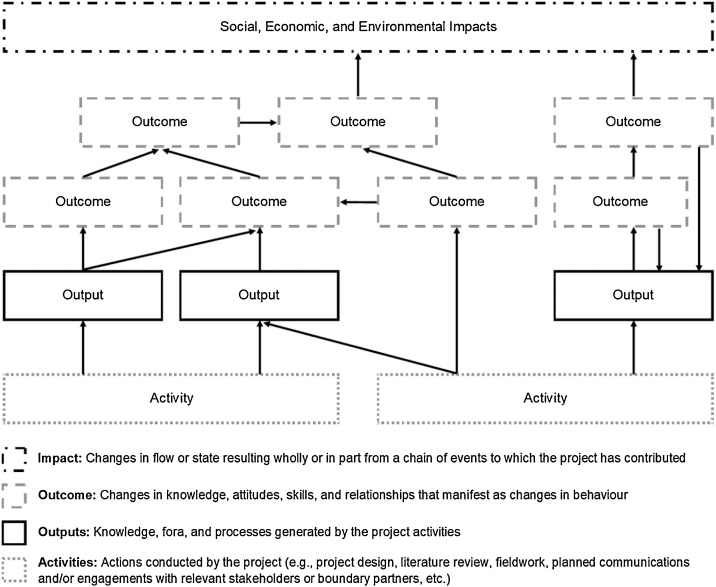
Sample theory of change flow diagram demonstrating logical flow of activities, outputs, outcomes, and impacts.

After the ToC workshop, the draft ToC model can be shared, reviewed, and refined in consultation with workshop participants and other stakeholders as appropriate. A narrative describing the causal logic behind the model should also be developed and subsequently verified by the workshop participants. It should be noted that if developed *ex ante*, it is important to revisit and iteratively revise the ToC throughout the project lifespan to ensure that activities and partnerships are sufficient and appropriate to achieve the intended outcomes. This can help ensure that new opportunities and emergent conditions are accounted for and effectively captured.

#### Developing an evidence table

The next step is to develop an evidence table. This can be done as part of the ToC workshop. The evidence table is a useful tool to plan, guide, and track data collection. The ToC serves as a guide to identify what data are needed to assess project design and implementation and outcome achievement to test each node in the ToC. The evidence table (Table 2; see [44] for a template) is organized by outcomes, and also documents the assumptions and indicators used to guide the outcome assessment. Indicators can help identify whether outcomes are happening and to what degree they are occurring. The framing we use captures a graduated scale of measures of success for each outcome. In practice, we ask workshop participants to identify what would: i) be expected at a minimum (expect to see); ii) indicate good success (like to see); and iii) indicate a high level of success (love to see) for each outcome. With this information, the data needed to evidence or refute project contributions to outcome achievement can be identified. Workshop participants can help direct the evaluators to identify what data already exist and what new data are required, as well as potential data sources (i.e., documents, surveys, key informants, media). Informant contact details (i.e., name, position/organization, phone number, and/or e-mail address) can be collected at this point in time, but can also be followed up post-workshop.

#### Data collection

Data are collected to evaluate actual outcomes against the ToC [40]. If an explicit ToC has been developed at the start of a project, relevant data can be collected at planned intervals during project implementation to monitor progress and inform adaptive management. In addition to what is identified in the evidence table to assess outcomes and understand the processes and mechanisms that contributed to outcome achievement, we recommend collecting data to understand project context, characterize project design and implementation, capture stakeholder perceptions of the research process and its outputs, determine use of knowledge produced by the project, and explore unexpected outcomes and alternative explanations to capture emergent phenomena characteristic of complex systems (see Appendix 2 which contains our interview guide for examples of how to collect this type of data).

The breadth of data needed for a comprehensive outcome evaluation often requires the use of mixed methods. We have used document review, bibliometrics/altmetrics, surveys, interviews, and focus groups to collect data. Documents such as project proposals, donor reports, meeting minutes, and e-mail correspondence have proven to be useful sources in addition to the tailored products produced by the project. Other forms of media, such as news articles, blog posts, and websites may also hold valuable evidence. Bibliometrics and altmetric data can be used to get an initial sense of whether project outputs have been used; however, the assessment should go a step further to uncover how project knowledge, products, or services have been used. Surveys can be useful tools to quickly collect broad or specific data from one or a range of actor groups, and can be adapted according to the data needs identified in the evidence table. Key informant interviews are an ideal way to collect the breadth and depth of data needed for the outcome evaluation. Key informants can include members of the research team, project participants, partners, collaborators, target audiences (e.g., representatives from government, NGOs, civil society organizations, communities affected by the topic, researchers, private sector, etc.), and academic or practitioner experts on the topic. Some informants may refer others who were not on the original list. Our interview guides (see Appendix 2 for an example; n.b. each guide would need to be tailored to the specific case study and set of guiding evaluation questions) were designed with a funnel approach, starting the discussion on broad topics to get information about the informant’s understanding of the problem context, key actors working within the system, ongoing developments and processes influencing the system, and decision-making and use of knowledge, before narrowing the focus to the informant’s knowledge and perceptions of the project and its contributions. Discussions within focus groups can use a similar structure to achieve the same ends.

#### Data management and analysis

We recommend using a qualitative analysis software (e.g., NVivo, MaxQDA, Coding Analysis Toolkit, etc.) to store, manage, transcribe, code, and analyze the data. Data should be coded deductively using a codebook that captures elements of project design and implementation as well as specific outcomes of the project (see Appendix 3 for an example codebook). Following the coding process, there are three analytical foci of the Outcome Evaluation approach to understand how and why outcomes were achieved: 1) factors of project design and implementation that supported or hindered outcome achievement; 2) informant perceptions of the types and extent of project contributions to outcome achievement; and 3) alternative explanations (i.e., factors and processes external to the project) for outcome achievement. We find the combination of these three lines of analyses powerful to uncover relationships between research approaches and contributions to change.

#### Data presentation

The Outcome Evaluation approach requires extensive data and analytical processing, which can make presentation of the findings complex and overwhelming for both evaluators and readers. To ease the reporting process, we recommend presenting the findings in a combination of narrative, graphical, and tabular form. Figures and tables will strengthen the discussion and provide a clearer and more digestible communication of findings. Providing answers to the overarching questions guiding the evaluation can help frame and organize reporting (see Appendix 1 for a generalized set of guiding evaluation questions applied in individual case study evaluations using the Outcome Evaluation approach).

The discussion should focus on whether and how the outcomes were achieved. This requires evidence that supports or refutes the logic underpinning the impact pathways within the ToC. Each step in the ToC should be assessed to determine if it was realized and present evidence of the extent that the project contributed to its realization. Some of this evidence may be perception-based, so it is important to present the information transparently between what has taken place, what and how actors believe a change has or will occur, and what is interpreted by the evaluators. To account for this, we include an assessment of the strength of evidence (i.e., low, medium, high; see right-hand column in Table 4) which is subjectively determined by the availability of evidence, reliability of evidence, triangulation of sources, and degree of interpretation required for the assessment.

Two tables are used to present the assessment of outcome achievement: a summary version is presented in the main body of the report and a full assessment is included in an appendix. The summary table (Table 3) comprises the results of the outcome assessment and the illustrative evidence used in the assessment. The results column lists the project outcomes identified in the ToC, and provides an assessment of the outcome achievement (i.e., achieved, partially achieved, not achieved, insufficient evidence) and the degree of project contribution (i.e., clear contribution of the project, indirect contribution of the project, unclear contribution of the project). A summary of the supporting evidence by outcome and source (e.g., interviews, documents, media) or indicators (either identified in the evidence table or proxy evidence indicative of the outcome’s achievement) is provided in one column. Another column is used to present how the outcome was achieved, looking at internal factors of the project, external factors of the system in which the project is situated, and the causal mechanisms or processes that supported the achievement of the outcome. The evidence presented in this table should be brief, as a more extensive analysis can be presented in the secondary table included in the appendix. For an example of how this data can be presented, see Belcher et al.’s ([25], Appendix 5) Supplementary materials.

**Table 3:** Example of a summary table presenting the outcome assessment, supporting evidence, and consideration of contextual factors and causal mechanisms affecting outcome achievement.

Results	Illustrative Evidence	
Outcome Assessment	Summary of supporting evidence for the assessment	Contextual factors and causal mechanisms affecting how the outcome was achieved
Outcome statement	Interviews: summary of evidence by actor group	Analysis of how the outcome was achieved (internal and external factors/mechanisms)
Assessment of achievement, assessment of degree of project contribution	Documents: summary of evidence	
	Media: summary of evidence	
	Indicators: evidence indicative of outcome achievement

**Table 4:** Example of expanded table presenting the outcome achievement, supporting evidence, degree of project contribution, and evidence rating for outcomes. Accompanying colour-coded legend used to indicate outcome achievement designation.

**Fig. 4:**
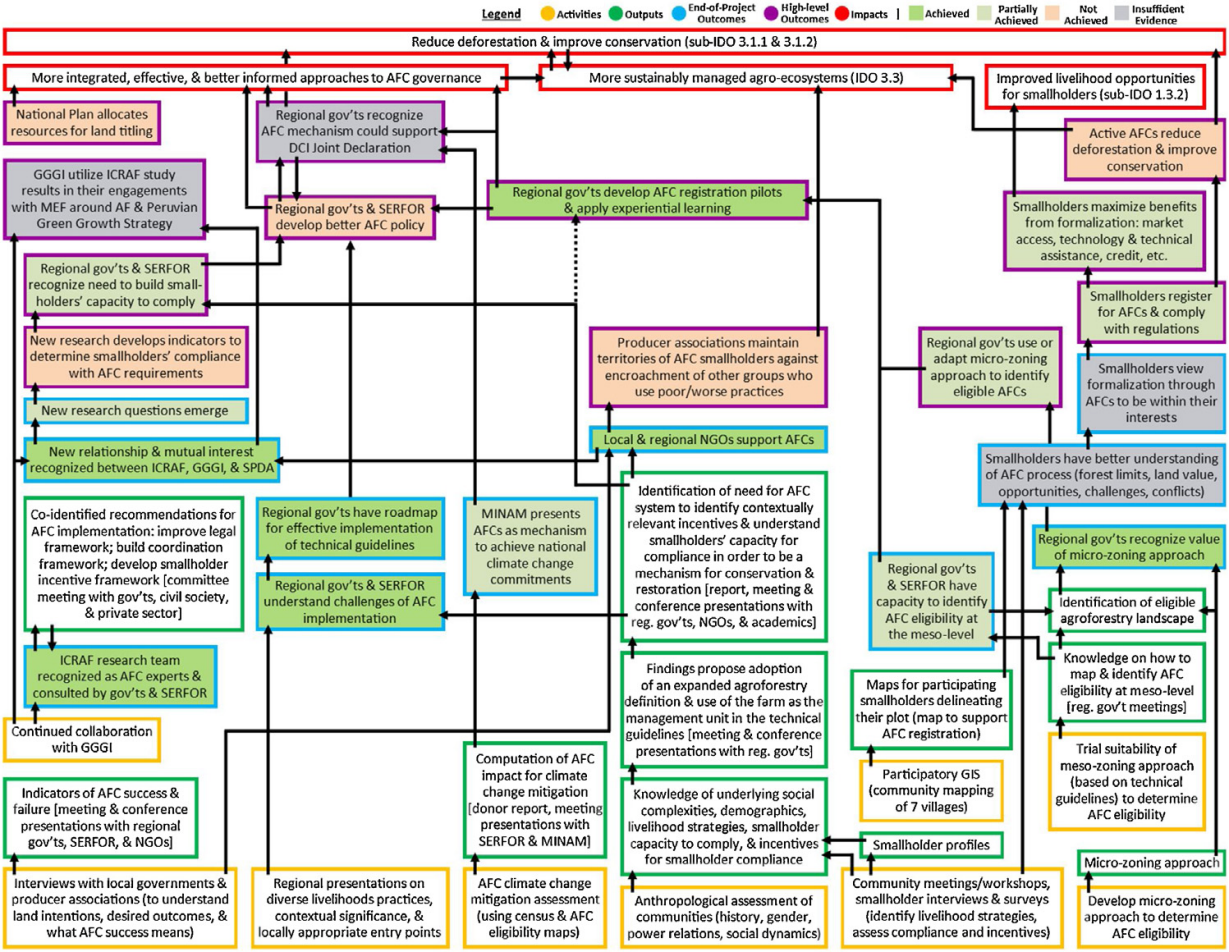
SUCCESS theory of change, with outcomes colour-coded to reflect the extent of outcome achievement [46].

We also compile an expanded outcome assessment table to be placed in an appendix (Table 4). Some of the content is similar to that included in the summary table, such as the list of outcomes under assessment by row, the assessment of each outcome’s achievement, how and why the outcome was achieved, and the assessment of the degree of project contribution. Additional information is integrated for a deeper analysis and transparent presentation of the outcome assessment. One column is dedicated to presenting evidence supporting the outcome’s achievement, such as direct quotations from informants (n.b. informants should remain anonymous; we recommend redacting identifiable information and using codes to represent the actor group), excerpts (e.g., from documents, websites, media), and citations (e.g., outputs, conferences, bibliometrics). Another column presents a rating of the strength of evidence available to make the assessment (i.e., low, medium, high), a justification for the designating assessment of the outcome’s achievement, and a legend used to indicate the outcome achievement (i.e., achieved, partially achieved, not achieved, insufficient evidence). The colour-coding in the legend corresponds with the colour-coding used in a figure of the ToC to visually illustrate the extent of outcome achievement (see Fig. 4). These components can be configured to the evaluators’ preference, as long as all are included in the outcome evaluation.

As mentioned, not only are these tables useful to present the achievement of outcomes, but this information can also be conveyed graphically in-text. Fig. 4 is an example ToC where the outcomes have been colour-coded to demonstrate their degree of achievement (other examples can be found in Appendix 6 of Belcher et al.’s [25] Supplementary materials).

As part of the outcome evaluation, is it important to understand the mechanisms of change and how they were influenced by the project’s design and implementation. In addition to the brief presentation in the summary table, we recommend dedicating part of the discussion to assess mechanisms by which outcomes were achieved. A table using symbols representing the mechanisms can help visualize this information (Table 5). The list of mechanisms is not exhaustive; these are the mechanisms identified from five project outcome evaluations conducted by the authors, and we welcome others to add to the list. This table also presents the mechanisms by impact pathway, which is useful to understand and potentially inform how other research projects intending to influence similar pathways could be approached (i.e., planning ways to leverage particular mechanisms over others in project design).

**Table 5:** Example of table illustrating the impact pathways and mechanisms leveraged in each case study presented in Belcher et al. [25].

In addition to the mechanisms, we explore and test alternative explanations for realized outcomes. This constitutes discussion of external factors and processes that either fully facilitated, partially contributed, or acted as a barrier to the achievement of outcomes. Our interview guide is designed to collect data about other actors, initiatives, and developments working in the same context as the research project under evaluation (see Appendix 2). Alternative explanations are used in lieu of a true counterfactual [14].

We recommend presenting the evaluation findings in draft form to project stakeholders for feedback, and discussing evidence and interpretations at a sense-making workshop with the project team, informants, and donors to provide validation, clarification, and feedback to the results. A sense-making workshop can be conducted in-person or hosted online. Feedback on results can be captured through a combination of comments made directly in the report, by e-mail, in the online chat, or by taking notes of the workshop discussion.

The time required to conduct an evaluation varies based on the scale of the project under investigation (i.e., project size, project duration, study location(s), etc.), availability of and accessibility to project documents, number and availability of informants, scope of the evaluation (i.e., purpose of the evaluation, objectives of the evaluation, donor requirements, whether additional inquiries are added to the evaluation), size of the evaluation team, involvement of external transcribers and translators, relative workload of the evaluators, and external review (i.e., feedback on reporting from participants and/or donors). In our outcome evaluations of international research-for-development projects, it has taken approximately one year on average from inception to final reporting. This includes delays involved with coordinating people across continents and time-zones, hiring and training interviewers internationally, and translating documents and interviews. The time required would be reduced with less complicating factors. The actual timing of an evaluation is also important to consider; this will be discussed below in reflection of the limitations of the approach.

#### Limitations

The Outcome Evaluation approach is not without limitations, but some can be accounted for through appropriate measures or adequate acknowledgement. Use of ToC and certain data collection methods are the main sources of limitations.

In *ex post* documentation of a ToC, it may not be possible to accurately capture the project team’s original intentions, expectations, and assumptions. Therefore, the retrospectively developed ToC may in part be a reflection of what actually occurred; for example, unexpected outcomes may be incorporated into the ToC that will be used as the analytical framework for the evaluation. While this would be considered a weakness in a summative evaluation, it is appropriate for a focus on learning. Moreover, as TDR theory encourages iterative adaptation to new knowledge and emergent conditions throughout a project, it is appropriate to use the most ‘up-to-date’ ToC to evaluate the project [25].

A project ToC often reflects ambitious targets. This is important to capture all potential project contributions, but it can create unrealistic expectations regarding the degree and scope of project contributions. The evaluation should be specific about the scope and scale of outcomes realized [45]. The concept of end-of-project outcomes, defined as realistic outcomes within the time and resources available, helps moderate expectations [22,25].

The timing of an outcome evaluation needs careful consideration. The full contributions of a research project may take time to be realized. An evaluation carried out too soon after project completion may miss changes that are still developing. Conversely, as time passes, key personnel may move on, memories fade, and other confounding events can occur. There is no perfect time for an evaluation as each research project’s context is different. An outcome evaluation should be considered a snapshot of a continual process. We have conducted outcome evaluations less than a year after the project’s official end-date and data were collected as project outputs were still being produced and disseminated [25,45].

It is also difficult for informants to recognize and identify project contributions, especially in contexts where there are other processes and projects operating with similar objectives [45]. It is inherently challenging for anyone to distinguish between the sources of knowledge and other influences; informants naturally find it difficult to attribute or make connections between the role of knowledge, a specific project, and a change process [22,34,45]. Informants often share impressions without concrete or specified evidence to support their perceptions [46,47]. As a result, informants may under- or overestimate a project’s contribution. Therefore, where possible, documents should be used to supplement and triangulate informant knowledge, but this requires that evidence is documented which is not always guaranteed or accessible [46].

As informants are primarily identified by members of the project team, there is a possibility for bias to be introduced to the process (i.e., gatekeeping, not identifying project ‘critics’ or actors with whom the project experienced conflict). This bias potential can be reduced by involving a range of project stakeholders as participants in the ToC workshop. Another solution would be to increase the sample size of informants, ensuring that there is adequate representation of all relevant stakeholder groups. This offers a triangulation of responses [45].

As discussed above, counterfactual reasoning is not possible in a single complex case. The Outcome Evaluation approach explicitly documents and tests alternative explanations for key outcomes using expert knowledge and empirical data in lieu.

Finally, the participatory approach used in the Outcome Evaluation method may not meet expectations of independence that some evaluation clients may demand. The involvement of the project team in the evaluation design aligns with the formative nature of the approach and is more conducive to learning [22,48,49]. Schneider et al. [49] discuss the advantages of engaging actors closely involved in the project under evaluation, as these actors are “deeply knowledgeable […] and intrinsically motivated to unravel [lessons] that allow them to become more transformative” (p.28) in future projects. It is important for the evaluators to reflect on potential biases and consciously maintain objectivity, with transparent documentation of methods, ToC, analysis, and interpretation of results so that readers can assess the process and not just the conclusions. Independent peer reviewers can be used to help scrutinize the evaluation.

## Declaration of Competing Interest

The authors declare that there are no conflicts of interest.
