# Supplementary material for: A refined method for theory-based evaluation of the societal impacts of research
Source: MethodsX. 2020 Jan 23;7:100788. doi: 10.1016/j.mex.2020.100788 (PMC6997623; doi:10.1016/j.mex.2020.100788)
Supplement: Supplementary file 1 [file mmc1.docx]

# Appendix 1. Generalized set of guiding evaluation questions used in the Outcome Evaluation approach.

The authors have applied the following set of guiding evaluation questions in individual case study outcome evaluations. The evaluation questions are organized to focus on assessments of outcome achievement and project design and implementation. These evaluation questions are aligned with the questions in the interview guide (see Appendix 2) and the codebook (see Appendix 3). The evaluation questions can be tailored to incorporate additional inquiries to accommodate donor requirements, specific evaluation objectives, or case study contexts.

***Research Outcome Evaluation:***

*To what extent and how were outcomes achieved?*

- *Were there any positive or negative unexpected outcomes from this project?*
- *Could the outcomes have been achieved in the absence of the project?*
- *Did the assumptions hold true?*
- *Are the higher-level changes likely to be realized?*

***Research Project Assessment:***

*What elements of the research design and implementation supported outcome achievements, and how?*

- *To what extent and how did the project engage effectively with relevant stakeholders?*
- *To what extent was/were the research process/findings sufficiently relevant to achieve the stated objectives?*
- *To what extent are target audiences aware of and using the project outputs? How are they using them?*
- *What lessons about effective research practice can be learned from this case study?*

# Appendix 2. Example Interview Guide used for the SUCCESS Project Outcome Evaluation (Claus, Davel, & Belcher, 2019)

*A) General questions (purpose to build trust & clarify the context)*

| **Main Question** | **Probes** | **Intent: What we are trying to find out**  **Do NOT ask these directly.** |
| --- | --- | --- |
| 1. What is your role within [organization], and what is your connection to agroforestry concessions? | - How long have you been doing this kind of work? - Try to find out their position/status in the community (how long they lived in the community, native or migrant, etc.) - How is your work related to the agroforestry issues? - What role have you and your organization played in the agroforestry concessions discussions/work, and for how long? - What is the intent of your organization in addressing agroforestry concessions policy and implementation issues? - How many people within your organization (what percentage of the organization) work on agroforestry concessions topics? | ***Decision-making power, familiarity with their own job/ organization & the relevance of the topic to their work***  The expertise of the person and his/her decision-making power (level of authority within the system in question, which is not necessarily formal power), including their role in decision-making. Knowledge about the organization/ position and its relevance to what they are doing.  The relevance of their work and the decision-making power /type of influence they may have on the topic of focus. |

*B) Recent and/or significant changes & players in agroforestry concessions issues*

| **Main Question** | **Probes** | **Intent: What we are trying to find out**  **Do NOT ask these directly.** |
| --- | --- | --- |
| 2. What are the main challenges around agroforestry concessions? | - What makes it/them challenges? | ***Personal expertise & perceptions on the topic of focus***  Interviewee’s knowledge level, understanding, and perceptions on the problems & issues relevant to the focus of the project – what do they think the problems are and how do they frame the problems. |
| 3. What have been the most important developments related to agroforestry concessions in Peru in the last five years? | - - In the discussions, events, ideas, institutions, policy, and/or practice?^[[1]](#footnote-1)^   - Why do you think these are important? | ***Understanding people’s perceptions of the situation and identifying possible changes in policy & practice.***  Understanding how issues (e.g., AFCs) are perceived and conceptualized by interviewees (this will allow for an overall characterization of the change process, including but not limited to how respondents think the project contributed. This will help construct narratives about alternative and/or supplementary ToCs.), range of various perspectives, and people’s understanding of the developments, causalities, & people’s values in relation to issues.  QAF: Rel1, Rel2, Rel3 |
| 4. Who are the key players in discussing, debating, and/or governance of agroforestry concessions? | - What role do government/academic/NGO/international/ private sector/communities play^[[2]](#footnote-2)^? - In what way have they (each) been influential? - Who does work related to the norms/regulations, implementation, facilitation? - Are there other influential persons or organizations that have a lot of influence in agroforestry concessions issues? | ***Understanding people’s perceptions of who is who in changing policy & practice.***  Getting an overview of who people consider as key actors in the process. This question will also provide insights about the power dynamics between the stakeholders (e.g. who’s got power over whom).  Here we want to identify the main actors that have been involved in the developments identified I the previous question. Be careful to not focus on the project and the role of the project until later.  QAF: Rel1, Rel3 |
| 5. What information/ knowledge or process/event has been the most influential in the discussion or media relating to agroforestry concessions in Peru? | - Who is promoting the information/knowledge or event in question? - In your opinion, has the information [what they mentioned] influenced policy and practice? How? Any specific examples? | ***Understanding what kind of knowledge is used in decision-making in general.***  Getting a better picture of what kind of knowledge & other factors are influencing agroforestry concessions decision-making and implementation, and where the ideas are coming from. More detailed information about possible changes in policy & practice because of new information/scientific knowledge.  QAF: Rel1, Rel2, Rel3 |

*C) Understanding links between knowledge sharing and decision-making processes (purpose to assess important sources of influence on policy and practice)*

| **Main Question** | **Probes** | **Intent: What we are trying to find out**  **Do NOT ask these directly.** |
| --- | --- | --- |
| 6. When doing work related to agroforestry concessions, where do you (or your organization) get knowledge you need to do your work? | - Probe to specify to understand what kind of information they mean, if needed - What kinds of information? - How is that information used (to create debate, to be used directly in policy formulation, to guide management decisions and implementation)? | ***Understanding what kind of knowledge is used in decision-making in general***  Getting a better picture of what kind of information is seen as important and/or used in decision-making (scientific or non-scientific)  QAF: Rel7, Eff2 |
| 7. Which of the following factors are the ones that influence the most your (personal and/or organization) decisions and position on agroforestry concessions? | \| - Political factors - Individual or organizational advocates - Scientific information/ research \| - Public opinion - Precedent in other jurisdictions - Global pressures/ influences \| \| --- \| --- \|  - Are there any additional factors? | ***Understanding what other aspects influence decision-making***  Understanding how people see decision-making situations, which aspects matter most in making changes in policy & practice, and how research findings matter in relation to other factors. |
| 8. Do you use scientific information in your work in relation to agroforestry concessions? | - How has it influenced or contributed to your work? - Where did you get that information? (Any specific events, publication, meetings, etc.) - Are there any barriers to using scientific information in this process? - What role do experts, advisory panels, researchers, among others play in providing information to your organization? - How can the role/engagement with experts, advisory panels, researchers for advancing your work in agroforestry concessions implementation be improved? | ***Understanding what the role of science is in decision-making***  Getting a better picture of the ways in which scientific knowledge is used by organisations, how they get the science they use, and what prevents them from basing their decision-making on scientific research findings.  QAF: Rel7, Eff2, Eff3 |

*D) ICRAF-related questions (purpose to assess outcome achievement and research influence on raising awareness and advancing understanding about agroforestry concessions implementation issues)*

| **Main Question** | **Probes** | **Intent: What we are trying to find out**  **Do NOT ask these directly.** |
| --- | --- | --- |
| 9. What do you know about work that ICRAF has done on agroforestry concessions?  (If they do not know ICRAF’s work, ask what they know about the work of partners on the topic) | [to non-partners]   - How did you hear about the project?   [to partners]   - When did you get involved in the SUCCESS project on agroforestry concessions? - How did you find the opportunities to participate and be involved? - What was your role? - How much time did you spend in work related to this project? - What was your contribution to this project? (e.g., Did you take part in meetings, workshops? Did you give recommendations to the project?) - Did you think that your input was taken into account? - What were the most important things you learnt? - Do you have any suggestions regarding how engagement and more meaningful participation can be improved? | ***Role & length of engagement with project partners***  Finding out to what extent the degree & length of engagement in the project may be associated with changes in policy & practice.  QAF: Rel3, Rel7, Cre7, Cre8, Leg1, Leg2, Leg3, Leg4, Eff2 |
| *[Ask 10-13 ONLY from partners & those who said they know ICRAF and the project]* | | |
| 10. Has the work of the SUCCESS project led by ICRAF contributed to or influenced your work on agroforestry concessions? | - If yes, how? Why? - Any positive/negative impact on policy/practices/awareness/ knowledge/capacity? - In what ways? - Any concrete examples? | ***ICRAF’s influence on their work (re the topic of focus)***  Please see some background above.  Finding out about linkages between ICRAF’s work and their work on the topic of focus*, and whether ICRAF has contributed to changes in policy & practice, but also to the debate, awareness in the topic, knowledge, capacity, or any other type of contributions.  Getting a sense whether the change is perceived as positive or negative.  QAF: Rel5, Eff1, Eff2, Eff3, Eff4 |
| 11. Has the SUCCESS Project had an influence on agroforestry concessions? | - If yes, why do you think it has? If no, why not? - In what ways? - Knowledge about agroforestry concessions issues/rights/ barriers/opportunities? - Attitudes about agroforestry concessions rights? - Technical options and skills related to agroforestry concessions? - Capacity to engage in the issues? - Relationships that affect agroforestry concessions implementation? - Have there been any negative outcomes of ICRAF’s work? If yes, please describe them. | ***Influence of ICRAF’s project on the topic of focus***  This question is ONLY asked IF the person has mentioned that they know about the project in question (or they are/were a research partner).  Finding out about the explicit outcomes/impacts of the project in question **anywhere** (in the world) that the interviewee knows of, not just within their own work/organization.  QAF: Rel5, Eff1, Eff2, Eff3, Eff4 |
| 12. What would have happened in the agroforestry concession debate and policy making in Peru had ICRAF not been working on this topic? | - Probe to clarify if needed (the role of the project in improving collaboration, social networks, participation, engagement) | ***Testing “zero hypothesis”***  Using a different angle to understand the true influence of ICRAF by asking what would be different had ICRAF not done its work.  QAF: Eff4 |
| 13. If ICRAF had more time and resources to work on this issue, what would you recommend to help improve their work on the issue? | - Any specific/concrete examples? - What could ICRAF or other organizations do to help address these challenges? | ***Feedback***  Hold to the end of the interview – if the interviewee starts talking about it at the beginning, please lead them back to any of the questions above and ask to return to the question.  This Q allows participants to give feedback to ICRAF and helps identify gaps/challenges, but we know many of the problems already and do not want to let this dominate/ mislead the main focus of the interview.  Use this opportunity to increase the depth of any previous answers by probing and relating this question to any other points informants raise – if/when appropriate.  QAF: Rel3, Rel5, Rel5, Rel7, Cre1, Leg3 (*many elements could come up here – will depend on respondent) |

*E) Closing Questions*

[partners]

| **Main Question** | **Probes** | **Intent: What we are trying to find out**  **Do NOT ask these directly.** |
| --- | --- | --- |
| [partners only]  14. How was your partnership experience in the SUCCESS Project led by ICRAF? | [partners only]   - Any examples of positive experiences/what was done well? Any promising practices? - How could we make the partnerships work even better in the future? What could have been done better? (diplomatic way to ask for negative experiences) | ***Personal experience & feedback***  Further details of the influence of the project on the personal level, possible additional aspects (re: knowledge translation).  Potential for improvement.  QAF: Rel7, Leg2, Leg3 |
| [all informants]  15. Is there anything else you think we should consider with regard to the role of research in the policy process and changing practice? | - Anything else you would like to add? | ***Closing***  Last remarks, things they might want to add that were not included, and closure. |

# Appendix 3. Example Codebook used in the SUCCESS Project Outcome Evaluation (Claus, Davel, & Belcher, 2019)

| *Code* | *Description* | *Comment* |
| --- | --- | --- |
| Alternative explanation(s) | Discussion of other reasons or factors not connected to the project (external) that may contribute to or affect the realization of outcomes. | Aligned with questions from interview guide on other developments, factors, and challenges. |
| Application | Any reference to possible practical applications resulting from the research (or any other related research in the region/topic). Include comments of whether participants have used or applied knowledge from the project (or another project/training) in their work, and how it changed practices. Include any indication of future intentions to apply or use knowledge in academic, policy, or practice contexts. | • Evaluation Research Question 2d: *Are the target audiences/stakeholders using the project’s outputs, and how are they using them?*  • Eff4. Practical application |
| Assumptions | Any reference to the project theory of change assumptions. These include: i) producing relevant information in a credible and timely manner will increase the uptake and use of research; ii) findings are logically connected, conceptually appropriate, and scientifically robust to align with target audience initiatives (fit to purpose); iii) engagement efforts were sufficient to build important relationships with allies to ensure continuity; iv) people pay attention to numbers (quantification) that give findings relevance; v) if we understand the enabling conditions to support agroforestry, success is more likely (option-by-context approach, tailored solutions); vi) changes to AFC implementation policy which accommodate smallholder heterogeneity will have a greater likelihood of improving smallholder livelihoods. | • Evaluation Research Question 2g: *Did the project theory of change assumptions hold true?* |
| Bias | Identification of possible sources of bias: researchers’ positions (education, gender, culture, discipline, etc.), sources of support, financing, collaborations, partnerships, research mandate, assumptions, goals, and bounds on research. Includes bias of any partner or relevant stakeholder. Includes biased comments. | • Leg1. Disclosure of perspective |
| Changes in attitude | Evidence of changes in attitudes. | • Evaluation Research Question 2: *To what extent and how were the intended outcomes of the ICRAF SUCCESS Project achieved?*  • Eff1. Builds social capacity |
| Changes in behaviour | Evidence of changes in behaviour. | • Evaluation Research Question 2: *To what extent and how were the intended outcomes of the ICRAF SUCCESS Project achieved?*  • Eff1. Builds social capacity |
| Changes in knowledge | Evidence of changes in knowledge or understanding. | • Evaluation Research Question 2: *To what extent and how were the intended outcomes of the ICRAF SUCCESS Project achieved?*  • Eff1. Builds social capacity  • Eff1. Contribution to knowledge |
| Changes in relationships | Evidence of changes in relationships. | • Evaluation Research Question 2: *To what extent and how were the intended outcomes of the ICRAF SUCCESS Project achieved?*  • Eff1. Builds social capacity |
| Changes in skills | Evidence of changes in skills or capacity. | • Evaluation Research Question 2: *To what extent and how were the intended outcomes of the ICRAF SUCCESS Project achieved?*  • Eff1. Builds social capacity |
| Collaboration | Any aspect related to collaboration (roles, responsibilities, decision-making structures). | • Leg2. Effective collaboration |
| Communication | Any aspects related to communication: verbal/oral, visual, written, channels of communication, timeliness, inclusiveness, appropriateness, etc. Includes any form of communication between actors in the system. | • Rel7. Effective communication |
| Competencies for research & policy-making | Comments on appropriate or necessary competencies for producing/ translating knowledge. Comments on appropriate or necessary competencies for incorporating knowledge into policy-making. Includes comments on existing competencies of the research team or actors in the system. | • Cre5. Adequate competencies (re: research team)  • Eff1. Builds social capacity (re: changes in capacity) |
| Context and problem | Discussion about the social and ecological characteristics of the context. This includes respondents’ perceptions of the relevance of the research in relation to other problems. Problem identification. | Aligns with questions in interview guide pertaining to challenges and developments (characterization of the context and the problems within that context).  • Rel1. Clearly defined socio-ecological context  • Rel2. Socially relevant research problem |
| Decision-making | Any data pertaining to decision-making done during the project, or influences on stakeholder decision-making. Include any reference to ‘policy’. | Aligns with questions in the interview guide pertaining to decision-making and knowledge |
| Dissemination & knowledge sharing | Information on how, where, and with whom the research was shared (planned or unexpected opportunities). | Code aspects of ‘knowledge translation’ and ‘brokering’.  • Evaluation Research Question 2c: *To what extent are target audiences aware of the project’s outputs?* |
| Engagement | Discussion of engagement with social actors or ecological factors. | • Evaluation Research Question 2b: *To what extent did the project engage effectively with relevant stakeholders?*  • Rel3. Engagement with problem context  • Leg3. Genuine and explicit inclusion |
| Ethics | Any mention of ethical or unethical aspects related to the research project – process, engagement, unintended outcomes, etc. | • Leg4. Research is ethical |
| Facilitating factors & barriers | Comments related to factors that facilitated/supported or obstructed the research process and its contributions. May include comments about time, funding, human resources, contextual factors, etc. This will include external and internal factors. | • Evaluation Research Question 3: *What lessons can be learned in regards to the project context and outcome achievements from this case study?*  • Cre4. Feasible research project |
| Gender & youth | Comments related to how the project integrated gender and youth considerations. | • Evaluation Research Question 1a: *How well did the project integrate gender and youth considerations?* |
| Knowledge & knowledge sources | Comments of where people get their knowledge and how they use it in their work. Comments of what type of knowledge/research people perceive to be credible or useful. | • Evaluation Research Question 2a: *To what extent was the science produced sufficiently relevant to achieve its aims?*  • Evaluation Research Question 2c: *To what extent are target audiences aware of the project’s outputs?* |
| Lessons | Comments related to project context, design, and implementation that supported the research or how it could be improved. | • Evaluation Research Question 3: *What lessons can be learned in regards to the project context and outcome achievements from this case study?* |
| Power | Any aspects reflecting power and power dynamics. |  |
| Reflection | Comments related to self-reflection in context of the research, including what the reflection led to (change in direction or the way things were done, who was included, etc.) that address research shortcomings and indicate adaptations. | Note: code may only be useful for researcher interviews (confined to members or collaborators of the research team)  • Cre11. Ongoing monitoring and reflexivity |
| Relevant actors | Identification and information pertaining to actors relevant to the context, whether they be direct participants in the research, actors within the context, actors working on issues/topics within the context/system, or boundary partners. |  |
| Research design | Discussion about objectives, research question(s), design, and methods. Comments on relevance, timeliness, and appropriateness of the research design. Identification of the specific focus or gap being researched and why it is being researched. Reference to research questions. Any discussion about objectives (aims, goals, outcome expectations, expected contributions, donor requirements, etc.). | • Evaluation Research Question 1: *How was the project designed and implemented to maximize knowledge translation?*  • Rel1. Clearly defined context  • Rel2. Socially relevant research problem  • Rel4. Explicit ToC  • Rel5. Relevant research objectives and design  • Rel6. App. project implementation  • Cre1. Broad preparation  • Cre2. Clear research problem definition  • Cre3. Objectives stated and met  • Cre4. Feasible research project  • Cre6. Approach fits purpose  • Cre7. Appropriate methods  • Cre10. Limitations stated |
| Social networks | Any reference to networks and connections between people or organizations that go beyond knowing about the other's existence. |  |
| Transferability & generalizability | Comments on perceptions or observation of other contexts where the findings or research process, approach, or methods can be applied. | • Cre9. Transferability/generalizability of findings |
| Trust | Comments related to relationships and trust. Also trust of researcher, findings, organizations, or other actors in the system. |  |
| Unexpected outcomes | Comments of other changes in knowledge, attitudes, skills, relationships, and/or behaviour resulting fully or in part from the research that were not identified by the research team. Can be positive or negative outcomes. | • Evaluation Research Question 4: *Were there any positive or negative unexpected outcomes from this project?* |
| *Case-specific Outcomes*  Outcomes were identified in the ToC workshop and are reflected in the ToC model.  • Eff4. Significant outcome | | |
| ICRAF research team recognized as AFC experts & consulted by governments & SERFOR | End-of-project outcome. | • Evaluation Research Question 2e: *Have the end-of-project outcomes been realized?* |
| ICRAF research team invited to formal committee formed to discuss AFCs | End-of-project outcome. | • Evaluation Research Question 2e: *Have the end-of-project outcomes been realized?* |
| Local & regional NGOs support AFCs | End-of-project outcome. | • Evaluation Research Question 2e: *Have the end-of-project outcomes been realized?* |
| Producer associations maintain territories of AFC smallholders against encroachment of other groups who use poor/ worse practices | High-level outcome. |  |
| New relationship & mutual interest recognized between ICRAF, GGGI, & SPDA | End-of-project outcome. | • Evaluation Research Question 2e: *Have the end-of-project outcomes been realized?* |
| GGGI utilize ICRAF study results in their engagements with MEF around agroforestry & Peruvian Green Growth Strategy | High-level outcome. |  |
| Regional governments & SERFOR understand challenges of AFC implementation | End-of-project outcome. | • Evaluation Research Question 2e: *Have the end-of-project outcomes been realized?* |
| Regional governments have a roadmap for effective implementation of technical guidelines | End-of-project outcome. | • Evaluation Research Question 2e: *Have the end-of-project outcomes been realized?* |
| Regional governments & SERFOR develop better AFC technical guidelines | High-level outcome. |  |
| MINAM presents AFCs as mechanism to achieve national climate change commitments | End-of-project outcome. | • Evaluation Research Question 2e: *Have the end-of-project outcomes been realized?* |
| Regional governments recognize value of micro-zoning approach | End-of-project outcome. | • Evaluation Research Question 2e: *Have the end-of-project outcomes been realized?* |
| Micro-zoning approach integrated into technical guidelines | High-level outcome. |  |
| Regional governments use & adapt micro-zoning approach to identify eligible AFCs | High-level outcome. |  |
| Regional governments & SERFOR have capacity to identify AFC eligibility at the meso-level | High-level outcome. |  |
| Regional governments develop AFC registration pilots | High-level outcome. |  |
| New research questions emerge | End-of-project outcome. | • Evaluation Research Question 2e: *Have the end-of-project outcomes been realized?* |
| New research develops indicators to determine smallholders’ compliance with AFC requirements | High-level outcome. |  |
| Regional governments & SERFOR recognize need to build smallholders’ capacity to comply | High-level outcome. |  |
| National Plan allocates resources for land titling | High-level outcome. |  |
| Regional governments recognize AFC mechanism could support DCI Joint Declaration | High-level outcome. |  |
| More integrated, effective, & better informed approaches to AFC governance | Impact. |  |
| More sustainably managed agro-ecosystems (IDO 3.3) | Impact. Alignment with IDO 3.3 (*More sustainably managed agro-ecosystems*). | • Evaluation Research Question 2f: *Are the changes in forestry practices likely to contribute to intended development outcomes (CGIAR IDO and sub-IDOs)?* |
| Smallholders have better understanding of AFC process (forest limits, land value, opportunities, challenges, conflicts) | End-of-project outcome. | • Evaluation Research Question 2e: *Have the end-of-project outcomes been realized?* |
| Smallholders engage in discussions around AFCs | End-of-project outcome. | • Evaluation Research Question 2e: *Have the end-of-project outcomes been realized?* |
| Smallholders equipped to judge whether to register for AFCs | End-of-project outcome. | • Evaluation Research Question 2e: *Have the end-of-project outcomes been realized?* |
| Smallholders more likely to view formalization through AFCs to be within their interests | End-of-project outcome. | • Evaluation Research Question 2e: *Have the end-of-project outcomes been realized?* |
| Smallholders register for AFCs | High-level outcome. |  |
| More smallholders comply with AFC requirements | High-level outcome. |  |
| Smallholders have access to technical assistance (e.g., technology, extension services) | High-level outcome. |  |
| Smallholders have access to legal land tenure | High-level outcome. |  |
| Active AFCs reduce deforestation & improve conservation | High-level outcome. |  |
| Reduce deforestation & improve conservation (sub-IDO 3.1.1 & 3.1.2) | Impact. Alignment with sub-IDO 3.1.1 (*Land, water and forest degradation minimized and reversed*). Alignment with sub-IDO 3.1.2 (*Enhanced conservation of habitats and resources*). | • Evaluation Research Question 2f: *Are the changes in forestry practices likely to contribute to intended development outcomes (CGIAR IDO and sub-IDOs)?* |
| Compliant smallholders are better able to access credit through national development banks | High-level outcome. |  |
| Smallholders maximize benefits from AFCs | Impact. |  |
| Improved livelihood opportunities for smallholders (sub-IDO 1.3.2) | Impact. Alignment with sub-IDO 1.3.2 (*Increased livelihood opportunities*). | • Evaluation Research Question 2f: *Are the changes in forestry practices likely to contribute to intended development outcomes (CGIAR IDO and sub-IDOs)?* |

1. All terminology should be adjusted & verbally explained so it is appropriate to each interviewee (please record any adaptations in the post-interview notes). [↑](#footnote-ref-1)
2. It is not necessary to ask all questions to every informant – the list merely illustrates what kind of information we are trying to find out. [↑](#footnote-ref-2)
